# Dr. W. T. Linn

**Published:** 1913-09

**Authors:** 


Dr. W. T. Linn died at his home near Pana, Ill., July 28. He was a native of Ohio. He had practiced medicine for over 75 years. He left 62 grandchildren and 53 great grandchildren, and was reputed to be the oldest man in the state, 108, but recent statements by relatives show that he was 98.

